# A Two-Stage VM Migration Framework for Power-Constrained Data Center Load Scheduling

**DOI:** 10.3390/s26134041

**Published:** 2026-06-25

**Authors:** Xiande Bu, Haixin Sun, Feng Tian, Xiaomin Li

**Affiliations:** 1 School of Communications and Information Engineering, Nanjing University of Posts and Telecommunications, Nanjing 210003, China; buxiande2@epri.sgcc.com.cn (X.B.); hxsun@xmu.edu.cn (H.S.); 2 State Grid Smart Grid Research Institute Co., Ltd., Beijing 102209, China; 3 School of Informatics, Xiamen University, Xiamen 316005, China; 4 Institute of Advanced Technology for Carbon Neutrality, Nanjing University of Posts and Telecommunications, Nanjing 210003, China; 2025050409@njupt.edu.cn

**Keywords:** data centers, information load scheduling, virtual machine migration, dynamic power constraints, multi-factor equilibrium optimization

## Abstract

With the rapid growth of data center (DC) energy consumption and the large-scale integration of renewable energy, DCs increasingly face time-varying power upper-bound constraints jointly shaped by grid power supply capability, renewable energy fluctuations, and demand response mechanisms. Meanwhile, DC power consumption exhibits a typical information-load-driven characteristic. The computing tasks hosted by virtual machines affect server-side IT power consumption through resource utilization states such as CPU, memory, disk I/O, and network I/O, and are further coupled with non-IT auxiliary power consumption from cooling, power distribution, and networking equipment. In such cyber–physical operation scenarios, physical-layer sensing data and hypervisor-level virtualization monitoring data jointly provide the state basis for power estimation, power warning, and migration decisions. To address the mismatch between dynamic power upper bounds and time-varying information loads, this paper investigates the information load scheduling problem under constrained power loads and proposes a two-stage virtual machine (VM) migration optimization framework. In the VM selection stage, a Multi-Factor Balanced (MFB) algorithm is designed. By introducing a warning-line trend model based on the arctangent function, MFB comprehensively considers resource utilization, power load variation trends, and service level agreement (SLA) violation levels to dynamically identify candidate VMs for migration. In the VM placement stage, a Multi-Factor Equilibrium Ant Colony Optimization (MFEACO) algorithm incorporating a Random Roulette Wheel (RRW) selection mechanism is proposed. By constructing normalized multi-dimensional equilibrium factors, MFEACO coordinates the trade-off among energy consumption, load balancing, and SLA violations. Simulation experiments are conducted on an improved CloudSim platform using real-world cluster trace data from Google and Alibaba. The results show that, while satisfying dynamic power constraints, the proposed MFB–MFEACO framework achieves a favorable comprehensive trade-off among energy consumption control, SLA violation suppression, and migration reduction. Compared with traditional heuristic methods and a power-constrained genetic algorithm baseline, the proposed framework demonstrates better dynamic adaptability and scheduling stability.

## 1. Introduction

The rapid development of cloud computing, big data, artificial intelligence, and the Internet of Things (IoT) has enabled modern sensor networks to generate large-scale, heterogeneous, and dynamically updated data [1]. Sensing data collected from industrial sensors, mobile terminals, smart meters, and various IoT devices generally rely on back-end data centers (DCs) for storage, processing, analysis, and intelligent decision support [2]. In this context, DCs are no longer only conventional computing and storage facilities; they have also become critical infrastructure nodes that connect physical sensing, information processing, and digital services [3,4]. However, the increasing dependence on DCs also leads to rapidly growing operating loads and energy costs, making energy consumption a primary challenge for their sustainable development.

This challenge has become more prominent as sensor-data-driven services continue to expand. The deployment scale and operating load of DCs are increasing continuously, resulting in a development pattern characterized by both rapid expansion and high energy consumption [5]. Industry reports indicate that the deployment scale of DC racks is growing at a compound annual growth rate of more than 30% [6]. According to the International Energy Agency (IEA) [7], the annual global electricity consumption of DCs has reached approximately 460 terawatt-hours (TWh) and is expected to exceed 1000 TWh in the future. Such high energy consumption increases operating costs, intensifies greenhouse gas emissions, and imposes additional pressure on power grids. Relevant projections further indicate that carbon emissions from DCs may reach 720 million tons by 2030 [8]. Therefore, reducing DC energy consumption while maintaining large-scale sensor data processing capability and service reliability has become a critical issue in the DC field. To address this issue, both academia and industry have investigated energy-saving technologies for DCs from different perspectives.

Existing studies on DC energy consumption optimization can be broadly classified into two categories. The first category focuses on improving the internal energy efficiency of DCs, mainly through power consumption modeling and server resource consolidation [9,10]. These methods usually regard a DC as a relatively independent computing system and primarily aim to improve server utilization, reduce idle resources, and achieve local load balancing. The second category improves DC energy efficiency from the perspective of energy supply and power-system coordination. With the widespread deployment of renewable energy sources such as wind and solar power, replacing conventional energy with renewable energy has become an important strategy for constructing green DCs [11]. Geographically distributed DCs can further exploit regional differences in electricity prices, renewable energy availability, and cooling conditions to perform information load management, thereby reducing operating costs and environmental impacts [12]. Meanwhile, virtualized computing units, such as virtual machines (VMs) and containers, have cross-regional migration capabilities, which provide a technical basis for the spatial redistribution of information loads [13]. In recent years, researchers have further explored the power flexibility of DCs from the perspectives of smart grid services, energy storage system configuration, and coordinated energy–computation task scheduling [14,15].

However, renewable energy generation is inherently volatile and intermittent, which may cause mismatches between grid-side power supply capability and DC information load demand [16]. To alleviate grid congestion and improve supply–demand coordination, mechanisms such as dynamic electricity pricing, demand response, and power load control have gradually been introduced into DC operation management [17]. Related studies in power systems also show that power scheduling, power quality enhancement, and robust power control generally need to deal with dynamic, uncertain, and strongly coupled constraints [18]. Under such conditions, the available power capacity of a DC may be jointly affected by grid supply capability, renewable energy output, and local power supply configuration [19]. Therefore, DC information load scheduling is no longer merely a problem of reducing total energy consumption. It must also ensure that the actual operating power of each DC remains within its currently available power capacity. When the available power capacity of a DC becomes more restrictive, or when its actual power demand approaches the power upper bound, VMs can be migrated across DCs to adjust the spatial distribution of information loads. In this way, part of the computing tasks can be transferred from power-constrained DCs to DCs with sufficient power margins and resource capacities.

Many studies have investigated VM consolidation, migration, and placement from different perspectives. Li et al. developed a VM consolidation algorithm based on dynamic load mean and multi-objective optimization to balance resource utilization and energy-saving performance [20]. In edge computing environments, Wang et al. investigated a lightweight VM scheduling approach to improve real-time service performance and minimize latency [21]. For the VM migration process, Ma et al. divided migration tasks into three distinct phases and introduced an adaptive dynamic threshold based on energy consumption levels together with a correlation-aware VM selection strategy to reduce overall energy consumption [22]. In addition, VM placement is essentially a large-scale discrete combinatorial optimization problem that requires comprehensive trade-offs among physical host capacity, resource utilization, energy consumption, quality of service, and migration decisions. Therefore, metaheuristic optimization algorithms have also attracted extensive attention. Alourani et al. [23] proposed a VM placement method combining a genetic algorithm and adaptive thresholds to reduce DC energy consumption and SLA violations by identifying overloaded and underloaded hosts. Gopu et al. [24] applied the NSGA-III multi-objective evolutionary algorithm to the VM placement problem in distributed clouds, jointly optimizing resource wastage, power consumption, and network transmission delay. Ashraf and Porres [25] designed a multi-objective ant colony system algorithm to construct VM migration plans, thereby improving physical machine consolidation and reducing migration operations. Aryania et al. [26] incorporated migration energy consumption into an ant-colony-system-based VM consolidation method to reduce the number of active physical machines, the number of migrations, and the total energy consumption.

Although the above studies have made progress in DC energy saving, VM consolidation, and multi-objective placement, several limitations remain in dynamic power-constrained scenarios. First, existing internal resource optimization methods mainly focus on server utilization or total energy consumption, while lacking explicit modeling of grid-side dynamic power upper bounds and their variation trends. Second, existing power-side scheduling studies mainly focus on energy supply optimization, demand response, or power system control, but pay insufficient attention to how external power constraints affect internal VM selection and placement decisions in DCs. Third, existing VM migration and metaheuristic placement methods are usually designed for static resource capacities, energy consumption optimization, or local load balancing, while insufficiently considering the coupling among physical-layer sensing data, virtualized operational traces, power load warning information, and SLA risks. Therefore, in DC scenarios subject to dynamic power constraints, it is still necessary to develop a collaborative VM selection and placement optimization method that can jointly account for changes in power constraints, resource load states, and service quality risks.

To address these issues, this paper investigates information load scheduling for geographically distributed DCs under dynamic power constraints and proposes a two-stage VM migration optimization framework that integrates trend-aware VM selection and multi-factor equilibrium VM placement. The main contributions of this paper are summarized as follows:

- A cyber–physical collaborative information load scheduling model is constructed for DCs under dynamic power constraints. The model jointly incorporates grid-side time-varying power upper bounds, distributed sensing and monitoring data, and VM resource utilization states into the scheduling process. In this way, conventional computing resource optimization is extended to a collaborative optimization problem driven by power constraints, physical state awareness, and information load migration.
- A power-trend-aware Multi-Factor Balanced (MFB) VM selection algorithm is designed. Unlike traditional methods that select VMs to be migrated only according to instantaneous CPU utilization, host load, or static thresholds, MFB uses an arctangent function to characterize the variation trends of the power load warning line and the resource utilization warning line. It further combines VM-level SLA violations, power consumption contribution, and resource occupation to construct a migration priority scoring function. This mechanism adapts VM selection according to whether the constraints become tighter or more relaxed, thereby reducing the risks of delayed migration and unnecessary migration.
- A Multi-Factor Equilibrium Ant Colony Optimization (MFEACO) algorithm is proposed for VM placement under dynamic power constraints. Unlike standard ACO methods that rely only on fixed heuristic information or a single energy consumption objective, MFEACO first constructs a feasible VM set for each target DC based on power constraints and resource capacity constraints. It then employs a Random Roulette Wheel (RRW) mechanism to preferentially select VMs with moderate resource demands. Subsequently, normalized factors related to load balancing, power consumption, warning-line spacing, and SLA violations are used to update pheromones, thereby improving the comprehensive equilibrium of VM placement decisions under constrained power loads.

## 2. System Model and Problem Formulation

### 2.1. DC Architecture Model

**Figure 1:**
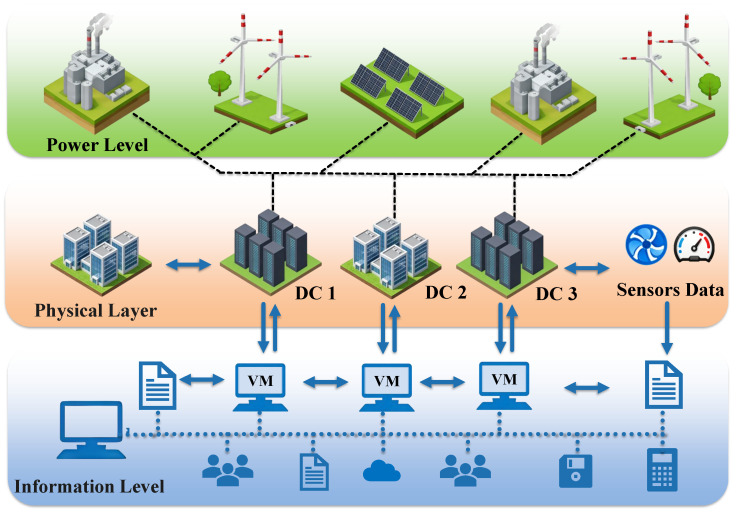
Cyber–physical collaborative information load scheduling architecture for multiple DCs under dynamic power constraints. The colored blocks denote the power, physical sensing, and information layers, and the arrows indicate power-supply coupling, sensing/monitoring data flow, and cross-DC information-load interactions.

Figure 1 presents the multi-DC information load scheduling architecture constructed in this paper. The architecture consists of the power layer, the physical sensing layer, and the information layer, and is used to describe the coupling relationship among dynamic power constraints, operational state monitoring, and VM migration scheduling. In the power layer, each DC is supplied by the main power grid and can also access local renewable energy sources such as photovoltaic power and wind power. Affected by grid supply capability, renewable energy output fluctuations, and demand response mechanisms, DCs need to satisfy time-varying power upper-bound constraints during operation. In the information layer, DCs are interconnected through networks, and the main information loads are composed of VMs or containers that carry computing tasks. Since VMs and containers can both be modeled as migratable computing units with multi-dimensional resource demands in cross-DC migration scheduling, this paper uses VMs to represent computing units in the following sections.

To support information load scheduling under dynamic power constraints, the physical sensing layer deploys distributed sensing and monitoring devices to continuously collect environmental and electrical state information during DC operation. Specifically, thermal sensors and cooling-system sensors are used to monitor cabinet inlet/outlet temperatures, airflow states, and cooling equipment operating states, thereby providing operational state support for analyzing variations in non-IT auxiliary power consumption. Smart meters, PDU monitoring devices, branch circuit meters, and UPS monitoring devices are used to collect electrical quantities such as active power, voltage, current, and regional power demand, thereby providing real-time data support for DC power consumption estimation, power load warning line construction, and dynamic power constraint judgment.

Meanwhile, hypervisor-level monitors are responsible for collecting operational traces in the information layer, including VM-level CPU utilization, memory usage, disk I/O, network I/O, VM deployment locations, resource allocation states, and migration records. The above physical-layer sensing data and virtualization-layer operational data jointly form a multi-dimensional operational state description during scheduling. Electrical monitoring data are used to characterize the real-time power demand of DCs and its constraint relationship with the power upper bound. VM resource utilization data are used to describe information load intensity and resource occupation states. Deployment and migration records are used to update the cross-DC load distribution state. Based on these state information, the scheduling algorithm further performs migration candidate VM selection and target DC placement optimization.

Based on the above virtualization-layer monitoring data, this paper abstracts a computing task as a multi-dimensional resource demand vector composed of CPU, memory, disk I/O, and network I/O. A computing task *T* is expressed as follows:

(1) $$T=\{u_{cpu},u_{mem},u_{disk},u_{net}\}$$

where $u_{cpu}$ denotes the CPU utilization of the task, $u_{mem}$ denotes the memory utilization, $u_{disk}$ denotes the disk I/O rate, and $u_{net}$ denotes the network I/O rate.

### 2.2. DC Energy Consumption Model

DC power consumption has an obvious information-load-driven characteristic. Unlike conventional electrical loads that are mainly determined by external electricity demand, the power demand of a DC is closely related to the computing tasks it hosts and the corresponding resource utilization states. The computing workload generated during VM operation directly affects server-side resource dimensions such as CPU, memory, disk I/O, and network I/O, and further influences server-side IT power consumption. Therefore, when constructing the DC energy consumption model, it is necessary to first characterize the mapping relationship between the power consumption of a single server and its multi-dimensional resource utilization. This paper adopts a multiple linear regression model to describe the power consumption $P_{s}$ of server *s*, which is expressed as:

(2) $$P_{s}=P_{min}+\beta_{1}u_{cpu}+\beta_{2}u_{mem}+\beta_{3}u_{disk}+\beta_{4}u_{net}$$

where $P_{min}$ denotes the idle power consumption of the server, and $\beta_{1}$, $\beta_{2}$, $\beta_{3}$, and $\beta_{4}$ denote the power consumption coefficients corresponding to CPU, memory, disk I/O, and network I/O, respectively.

Therefore, the overall server power consumption $P_{server}$ is obtained by summing the power consumption of all servers:

(3) $$P_{server}=\sum_{s\in S}P_{s}$$

In addition to servers, DC power consumption also includes cooling equipment, power conditioning equipment, networking equipment, and lighting equipment. Since calculating the power consumption of these devices individually is highly complex, this paper adopts a proportional method that links the power consumption of these components to server power consumption [27]. The total power consumption $P_{DC}$ of the entire DC is expressed as:

(4) $$P_{DC}=\left(1+\delta \right)P_{server}$$

where δ denotes the proportional factor between the power consumption of other equipment and server power consumption.

### 2.3. Problem Formulation for Information Load Scheduling

Consider a system consisting of *n* DCs, where four types of resources are mainly evaluated: CPU utilization ($u_{cpu}$), memory utilization ($u_{mem}$), disk I/O rate ($u_{disk}$), and network I/O rate ($u_{net}$). The DC resources can be defined as the following matrix:

(5) $$R={\left[R_{1},R_{2},\ldots ,R_{n}\right]}^{T},\;R_{j}=\left[R_{j}^{cpu},R_{j}^{mem},R_{j}^{disk},R_{j}^{net}\right]$$

To avoid symbol confusion, this paper uses R to denote the set of resource types:

(6) R={cpu,mem,disk,net}

The computing task *T* runs on VMs. The resource demand of VM *i* at time *t* is denoted by vector $V_{i}\left(t\right)$, which is expressed as:

(7) $$V_{i}\left(t\right)=\left\{V_{i}^{cpu}\left(t\right),V_{i}^{mem}\left(t\right),V_{i}^{disk}\left(t\right),V_{i}^{net}\left(t\right)\right\}$$

When VMs are scheduled across the network, the cumulative resource allocation of each target DC *j* at any time *t* must strictly satisfy its physical capacity constraints. Specifically, the total amount of resources requested by all VMs allocated or migrated to DC *j* must not exceed its capacity:

(8) $$\left\{\begin{array}{cc} \sum_{i\to j}V_{i}^{\mathrm{cpu}}\left(t\right) & \leq R_{j}^{\mathrm{cpu}}\left(t\right)\\ \sum_{i\to j}V_{i}^{\mathrm{mem}}\left(t\right) & \leq R_{j}^{\mathrm{mem}}\left(t\right)\\ \sum_{i\to j}V_{i}^{\mathrm{disk}}\left(t\right) & \leq R_{j}^{\mathrm{disk}}\left(t\right)\\ \sum_{i\to j}V_{i}^{\mathrm{net}}\left(t\right) & \leq R_{j}^{\mathrm{net}}\left(t\right) \end{array}\right.$$

Under constrained power loads, the key problem of information load scheduling is that different DCs face different dynamic power upper bounds $LP_{j}\left(t\right)$ at time *t*, while the time-varying characteristics of VM workloads continuously change the actual power demand $P_{DC_{j}}\left(t\right)$ and resource occupation states of each DC. When the power demand of a DC approaches or exceeds its available power upper bound, or when its multi-dimensional resource utilization enters a stressed state, the original VM placement scheme may no longer satisfy power constraints, resource capacity constraints, and service quality requirements simultaneously. Therefore, this paper readjusts the spatial distribution of information loads through cross-DC VM migration, so that some computing tasks are transferred from power-constrained or resource-stressed DCs to DCs with remaining power margins and resource capacities. In this way, potential power violations, resource overload, load imbalance, and QoS/SLA degradation risks can be alleviated.

Based on the above problem, this paper defines information load scheduling under constrained power loads as follows: under the premise of satisfying multi-dimensional resource capacity constraints and dynamic hard power constraints, the placement relationship of VMs in the candidate DC set DClist is determined, so that the total operating energy consumption of the system during the scheduling period is minimized. Since the simulation adopts discrete scheduling periods, the total operating energy consumption of the system can be expressed as the sum of the product of power and scheduling interval over all scheduling periods:

(9) $$\min\sum_{t\in T}\sum_{j\in DClist}P_{DC_{j}}\left(t\right)∆t$$

subject to:

(10) $$P_{DC_{j}}\left(t\right)\leq LP_{j}\left(t\right),\;\forall j\in DClist,\;\forall t\in T$$

where T denotes the discrete time set within the scheduling period, ∆t denotes the time interval between two adjacent scheduling moments, $P_{DC_{j}}\left(t\right)$ denotes the steady-state operating power of DC *j* at time *t*, and $LP_{j}\left(t\right)$ denotes the dynamic hard power upper bound available to DC *j* at time *t*.

In the above model, $P_{DC_{j}}\left(t\right)$ denotes the steady-state operating power consumption of DC *j* under a given VM placement state, which is mainly determined by server-side IT loads and their associated auxiliary power consumption, as described in Equations (2)–(4). Therefore, Equations (9) and (10) establish the constraint relationship between DC operating power consumption after VM re-placement and the dynamic power upper bound, and are used to evaluate the feasibility and energy consumption performance of scheduling schemes under constrained power loads.

## 3. Information Load Scheduling Under Constrained Power Loads

Based on the problem definition in Section 2.3, this paper further constructs a two-stage VM migration scheduling framework, including the selection of VMs to be migrated and VM placement. The first stage identifies migration candidate VMs from power-constrained or resource-stressed DCs, so as to effectively relieve the current operating pressure. The second stage selects target DCs with remaining power margins and resource capacities for the candidate VMs under the premise of satisfying power and resource constraints.

### 3.1. Selection of the Set of VMs to Be Migrated

The objective of selecting the set of VMs to be migrated is to choose migration candidate VMs from power-constrained or resource-stressed DCs, thereby relieving the power and resource pressure of source DCs and improving the feasibility of subsequent placement optimization.

This paper uses the power load warning line and the resource utilization warning line to characterize the stress levels of power consumption and resources, respectively. When the power consumption or resource occupation of a DC exceeds the specified warning line, the DC is regarded as being in an overloaded state, and VM migration is required for energy efficiency optimization. Based on the load balancing principle, and considering factors such as the power load warning line trend, VM utilization, and VM SLA violation degree, this paper proposes a Multi-Factor Balanced (MFB) algorithm for selecting the set of VMs to be migrated.

It should be noted that this paper distinguishes the dynamic hard power upper bound from the power warning line. $LP_{j}\left(t\right)$ denotes the dynamic hard power upper bound of DC *j* at time *t*, and the scheduled operating power must satisfy $P_{DC_{j}}\left(t\right)\leq LP_{j}\left(t\right)$. $WL_{j}^{P}\left(t\right)$ denotes the power warning line used to proactively trigger migration, whose value is lower than the hard upper bound:

(11) $$WL_{j}^{P}\left(t\right)=\alpha_{P}LP_{j}\left(t\right)$$

Here, $LP_{j}\left(t\right)$ is the power constraint boundary that cannot be violated, while $WL_{j}^{P}\left(t\right)$ is a safety warning threshold for proactive migration decisions.

Similarly, this paper uses $W_{j}^{r}\left(t\right)$ to denote the resource utilization of DC *j* in resource dimension *r*:

(12) $$W_{j}^{r}\left(t\right)=\frac{\sum_{v\in DC_{j}}V_{alloc,v}^{r}\left(t\right)}{R_{j}^{r}\left(t\right)},\;r\in R$$

where $R_{j}^{r}\left(t\right)$ denotes the physical resource capacity of DC *j* in resource dimension *r*.

Considering that DC operation may be affected by temperature states, cooling conditions, power distribution states, and virtualization-layer resource allocation states, this paper uses ${\tilde{R}}_{j}^{r}\left(t\right)$ to denote the dynamically available resource capacity of DC *j* in resource dimension *r*. This variable can be determined by the operating states jointly reflected by thermal sensors, cooling-system sensors, smart meters, PDU and UPS monitoring devices, and hypervisor-level resource monitoring data, and it satisfies:

(13) $$0<{\tilde{R}}_{j}^{r}\left(t\right)\leq R_{j}^{r}\left(t\right),\;r\in R$$

On this basis, the resource utilization warning line $WL_{j}^{r}\left(t\right)$ is defined as:

(14) $$WL_{j}^{r}\left(t\right)=\alpha_{r}\frac{{\tilde{R}}_{j}^{r}\left(t\right)}{R_{j}^{r}\left(t\right)},\;0<\alpha_{r}<1,\;r\in R$$

where ${\tilde{R}}_{j}^{r}\left(t\right)$ is the dynamic available resource upper bound under operational state constraints.

When $P_{DC_{j}}\left(t\right)>WL_{j}^{P}\left(t\right)$ or there exists a resource dimension satisfying $W_{j}^{r}\left(t\right)>WL_{j}^{r}\left(t\right)$, DC *j* is determined to trigger the selection of migration candidate VMs.

The VM selection metric first characterizes the relative SLA violation degree of a single VM. For VM *v*, if it is located in DC *j*, its SLA violation value at time *t* is defined as:

(15) $$SLAv_{v}\left(t\right)=\sum_{r\in R}w_{r}{\left[\frac{V_{v}^{r}\left(t\right)-V_{alloc,v}^{r}\left(t\right)}{R_{j}^{r}\left(t\right)}\right]}_{+}$$

where $w_{r}$ denotes the weight of resource dimension *r*, $V_{v}^{r}\left(t\right)$ denotes the amount of resource *r* requested by VM *v* at time *t*, $V_{alloc,v}^{r}\left(t\right)$ denotes the amount of resource *r* allocated to VM *v* at time *t*, $R_{j}^{r}\left(t\right)$ denotes the physical capacity of the DC *j* where VM *v* is located in resource dimension *r*, and ${\left[\cdot \right]}_{+}=\max\left(\cdot ,0\right)$ denotes the positive-part function. By dividing by $R_{j}^{r}\left(t\right)$, the violation amounts under different resource dimensions are converted into relative violation degrees, thereby avoiding the direct summation of indicators with different physical units, such as CPU, memory, disk I/O, and network I/O.

Furthermore, the overall SLA violation degree of DC $DC_{j}$ can be obtained by accumulating the violation values of its internal VMs:

(16) $$SLAv_{DC_{j}}\left(t\right)=\sum_{v\in DC_{j}}SLAv_{v}\left(t\right)$$

Next, the algorithm introduces the warning line trend, namely the slope, to determine whether to migrate VMs with high resource/power utilization or those with low resource/power utilization. If the slope of the warning line is negative, VMs with high resource occupation are migrated; otherwise, VMs with low resource occupation are migrated. This method can effectively reduce the overload frequency of DCs and the total number of migrations. The warning line slopes are calculated as follows:

(17) $$m_{j}^{P}=\arctan\left(\frac{WL_{j}^{P}\left(t_{2}\right)-WL_{j}^{P}\left(t_{1}\right)}{∆t}\right)$$

(18) $$m_{j}^{r}=\arctan\left(\frac{WL_{j}^{r}\left(t_{2}\right)-WL_{j}^{r}\left(t_{1}\right)}{∆t}\right)$$

where $WL_{j}^{P}\left(t_{1}\right)$ and $WL_{j}^{P}\left(t_{2}\right)$ denote the power load warning line values of DC *j* at two consecutive scheduling moments $t_{1}$ and $t_{2}$, respectively. $WL_{j}^{r}\left(t_{1}\right)$ and $WL_{j}^{r}\left(t_{2}\right)$ denote the warning line values of resource *r* in DC *j* at the corresponding moments, respectively. $∆t=t_{2}-t_{1}$ denotes the time interval between the two moments.

To enable the power consumption factor to participate in the migration priority ranking of individual VMs, this paper further estimates the marginal contribution of VM *v* to server-side power consumption:

(19) $$P_{v}\left(t\right)=\beta_{1}V_{v}^{cpu}\left(t\right)+\beta_{2}V_{v}^{mem}\left(t\right)+\beta_{3}V_{v}^{disk}\left(t\right)+\beta_{4}V_{v}^{net}\left(t\right)$$

where $P_{v}\left(t\right)$ denotes the approximate contribution of VM *v* to server power consumption under its current resource request state. This term is consistent with the multi-dimensional resource power consumption coefficients in Equation (2), and is used to distinguish the power consumption impacts of different candidate VMs in the VM selection stage.

To avoid dimensional inconsistency among different evaluation indicators, this paper adopts a unified normalization operator to normalize the SLA violation degree, VM power contribution, and VM resource allocation terms before calculating the migration priority score. For any non-negative evaluation indicator x(t), its normalized form is defined as:

(20) $$\hat{x}\left(t\right)=N\left(x\left(t\right)\right)=\frac{x(t)}{x^{ref}\left(t\right)}$$

where $x^{ref}\left(t\right)$ denotes the reference scale corresponding to indicator x(t). For the VM selection scoring function, the reference values of $SLAv_{v}\left(t\right)$ and $P_{v}\left(t\right)$ are taken as the maximum SLA violation value and the maximum VM power contribution value in the current candidate VM set, respectively. The reference value of the resource term $V_{alloc,v}^{r}\left(t\right)$ is taken as the physical capacity $R_{j}^{r}\left(t\right)$ of the corresponding resource dimension. After normalization, evaluation indicators with different physical dimensions are converted into dimensionless quantities.

To characterize the influence of warning line variation trends on the VM selection direction, this paper further introduces the power direction factor $d_{P}\left(t\right)$ and the resource direction factor $d_{r}\left(t\right)$:

(21) $$d_{P}\left(t\right)=\left\{\begin{array}{cc} -1, & m_{j}^{P}\leq 0\\ 1, & m_{j}^{P}>0 \end{array}\right.$$

(22) $$d_{r}\left(t\right)=\left\{\begin{array}{cc} -1, & m_{j}^{r}\leq 0\\ 1, & m_{j}^{r}>0 \end{array}\right.$$

where $m_{j}^{P}$ and $m_{j}^{r}$ denote the power load warning line slope and resource utilization warning line slope of DC *j*, respectively. When the warning line slope is negative, it indicates that the available power or resource constraint tends to tighten. In this case, the direction factor takes a negative value, so that VMs with higher power contribution or resource occupation obtain lower migration priority scores and are preferentially migrated out. When the warning line slope is positive, it indicates that the constraint tends to relax. In this case, the direction factor takes a positive value, so that VMs with lower resource occupation are more likely to be selected, thereby avoiding unnecessary large-scale migration overhead.

Based on the normalized indicators and direction factors, the VM migration priority score is defined as:

(23) $$S_{v}\left(t\right)=-w_{slav}{\hat{SLAv}}_{v}\left(t\right)+d_{P}\left(t\right)w_{P}{\hat{P}}_{v}\left(t\right)+\sum_{r\in R}d_{r}\left(t\right)w_{r}{\hat{V}}_{alloc,v}^{r}\left(t\right)$$

where $w_{slav}$, $w_{P}$, and $w_{r}$ denote the weights of SLA violation degree, VM power contribution, and resource occupation, respectively. ${\hat{SLAv}}_{v}\left(t\right)$, ${\hat{P}}_{v}\left(t\right)$, and ${\hat{V}}_{alloc,v}^{r}\left(t\right)$ are all dimensionless normalized indicators. Since the migration objects in this paper come from power-constrained or resource-stressed DCs, VMs with higher current SLA violation degrees should be migrated out with higher priority. Therefore, the SLA term adopts a negative sign, so that it obtains a higher migration priority under the minimization rule of $S_{v}\left(t\right)$. A smaller $S_{v}\left(t\right)$ indicates that VM *v* is more suitable to be selected as a migration candidate in the current scheduling interval.

The detailed description of the MFB selection algorithm is presented in Algorithm 1.

### 3.2. VM Placement Strategy

The objective of the VM placement stage is to assign the migration candidate VMs obtained from the MFB stage to appropriate target DCs under the premise of satisfying dynamic power constraints and resource capacity constraints. Unlike traditional VM placement methods that mainly perform greedy selection according to a single energy increment or host utilization metric, the proposed MFEACO method regards VM placement as a combinatorial optimization problem affected by multi-dimensional constraints. In this process, the available power margin, remaining resource capacity, and historical SLA state of target DCs all affect the final placement results, while the number of migrations serves as an important metric for evaluating migration overhead.
**Algorithm 1** MFB Selection Algorithm**Input:** 
DClist: set of DCs triggering migration; *V*: VM set; $V_{alloc}$: VM resource allocation state; LP: dynamic hard power limit; WLP: power load warning line; $WL^{r}$: resource utilization warning line; R: set of resource types; $w_{slav}$: SLA violation weight; $w_{P}$: power contribution weight; $w_{r}$: resource weight.**Output:** 
vmsToMigrate: set of VMs to be migrated.1:Initialize empty set vmsToMigrate←Ø2:**for** each $DC_{j}\in DClist$ **do**3:   Initialize candidate set $C_{j}\leftarrow \left\{v|v\in DC_{j}\right\}$4:   Initialize the temporary state of $DC_{j}$ according to the current scheduling state5:   **while** $(P_{DC_{j}}\left(t\right)>WLP_{j}\left(t\right)$ **or** $\exists r\in R,\;W_{j}^{r}\left(t\right)>WL_{j}^{r}\left(t\right))$ **and** $C_{j}\neq Ø$ **do**6:      Calculate the power warning-line slope $m_{j}^{P}$ using Equation (17):7:       $m_{j}^{P}\leftarrow \arctan\;\left(\frac{WLP_{j}\left(t_{2}\right)-WLP_{j}\left(t_{1}\right)}{\ldots t}\right)$8:       Calculate the power direction factor $d^{P}\left(t\right)$ using Equation (21):9:       $d^{P}\left(t\right)\leftarrow -1$ if $m_{j}^{P}\leq 0$; otherwise $d^{P}\left(t\right)\leftarrow 1$10:    **for** each r∈R **do**11:        Calculate the resource warning-line slope $m_{j}^{r}$ using Equation (18):12:        $m_{j}^{r}\leftarrow \arctan\;\left(\frac{WL_{j}^{r}\left(t_{2}\right)-WL_{j}^{r}\left(t_{1}\right)}{\ldots t}\right)$13:        Calculate the resource direction factor $d^{r}\left(t\right)$ using Equation (22):14:        $d^{r}\left(t\right)\leftarrow -1$ if $m_{j}^{r}\leq 0$; otherwise $d^{r}\left(t\right)\leftarrow 1$15:     **end for**16:     **for** each $v\in C_{j}$ **do**17:        Calculate the SLA violation degree $SLAv_{v}\left(t\right)$ using Equation (15):18:        $SLAv_{v}\left(t\right)\leftarrow \sum_{r\in R}w_{r}{\left[\frac{V_{v}^{r}\left(t\right)-V_{alloc,v}^{r}\left(t\right)}{R_{j}^{r}\left(t\right)}\right]}^{+}$19:        Calculate the VM power contribution $P_{v}\left(t\right)$ using Equation (19):20:        $P_{v}\left(t\right)\leftarrow \beta_{1}V_{v}^{cpu}\left(t\right)+\beta_{2}V_{v}^{mem}\left(t\right)+\beta_{3}V_{v}^{disk}\left(t\right)+\beta_{4}V_{v}^{net}\left(t\right)$21:        Normalize $SLAv_{v}\left(t\right)$, $P_{v}\left(t\right)$, and $V_{alloc,v}^{r}\left(t\right)$ using Equation (20):22:        $\hat{x}\left(t\right)\leftarrow N\left(x\left(t\right)\right)=\frac{x(t)}{x_{ref}\left(t\right)},\;x\in \left\{SLAv_{v},P_{v},V_{alloc,v}^{r}\right\}$23:        Calculate the migration priority score $S_{v}\left(t\right)$ using Equation (23):24:        $S_{v}\left(t\right)\leftarrow -w_{slav}{\hat{SLAv}}_{v}\left(t\right)+d^{P}\left(t\right)w_{P}{\hat{P}}_{v}\left(t\right)+\sum_{r\in R}d^{r}\left(t\right)w_{r}{\hat{V}}_{alloc,v}^{r}\left(t\right)$25:     **end for**26:     Select the VM with the minimum migration priority score:27:     $v^{*}\leftarrow \arg\min_{v\in C_{j}}S_{v}\left(t\right)$28:     $vmsToMigrate\leftarrow vmsToMigrate\cup \{v^{*}\}$29:     $C_{j}\leftarrow C_{j}\setminus \left\{v^{*}\right\}$30:     Remove $v^{*}$ from the temporary state of source data center $DC_{j}$31:     Update $P_{DC_{j}}\left(t\right)$ and $W_{j}^{r}\left(t\right)$ according to the updated temporary state32:   **end while**33:**end for**34:**return** vmsToMigrate

The basic idea of MFEACO is as follows. First, the pheromone matrix between VMs and DCs and the pheromone evaporation rate are initialized. Then, in each iteration, a feasible VM set satisfying the constraints is constructed for each candidate target DC, and VMs to be placed are selected from this set through the RRW mechanism. After one round of placement is completed, the algorithm updates the pheromone concentration according to the multi-factor equilibrium evaluation results related to energy consumption, load balancing, warning-line spacing, and SLA violations. After multiple iterations, the pheromone gradually concentrates on VM–DC mapping relationships with better comprehensive performance, thereby obtaining an approximately optimal placement scheme.

#### 3.2.1. Random Roulette Wheel VM Selection

In the MFEACO placement process, the RRW mechanism is used to determine which migration VM should be preferentially accepted by each candidate target DC in the current iteration. If a greedy strategy is directly used to select VMs with the largest resource occupation or the highest energy consumption contribution, it may lead to excessive migration overhead or resource imbalance in the target DC. If completely random selection is adopted, the pheromone experience accumulated in historical iterations cannot be effectively utilized. Therefore, this paper combines VM resource occupation equilibrium with ant colony pheromone concentration, and constructs a roulette-wheel selection mechanism with random disturbance to achieve a trade-off between exploration and exploitation.

For a given target DC, the feasible VM set $\Omega_{DC}\left(t\right)$ is first constructed according to power constraints and resource constraints:

(24) $$\Omega_{\mathrm{DC}}\left(t\right)=\left\{v\in \mathrm{vmsToMigrate}\left|\begin{array}{c} P_{\mathrm{DC}}\left(t|v\right)\leq LP_{\mathrm{DC}}\left(t\right),\\ \sum_{u\in \mathrm{DC}}V_{u}^{r}\left(t\right)+V_{v}^{r}\left(t\right)\leq R_{\mathrm{DC}}^{r}\left(t\right),\;\forall r\in R \end{array}\right.\right\}$$

where $P_{DC}\left(t|v\right)$ denotes the steady-state operating power of the target DC after VM *v* is placed into it, and $LP_{DC}\left(t\right)$ denotes the dynamic hard power upper bound of the target DC. $\sum_{u\in DC}V_{u}^{r}\left(t\right)$ denotes the total resource demand of the VMs already placed in the target DC in resource dimension *r*, and $V_{v}^{r}\left(t\right)$ denotes the resource demand of candidate migration VM *v* in resource dimension *r*. Only VMs that simultaneously satisfy the dynamic hard power constraint and multi-dimensional resource capacity constraints enter the subsequent RRW selection process.

Then, the RRW mechanism calculates the expected value $E_{v}\left(t\right)$ of each VM in the feasible set:

(25) $$E_{v}\left(t\right)=\sum_{r\in R}w_{r}\left(1-\left|\theta_{r}-R_{v}^{r}\left(t\right)\right|\right)+\eta \xi_{v}\left(t\right)$$

where $w_{r}$ denotes the weight of resource dimension *r*, $R_{v}^{r}\left(t\right)$ denotes the normalized utilization of resource *r* by VM *v* at time *t*, and $\theta_{r}$ denotes the equilibrium utilization reference value of resource dimension *r*. Since $R_{v}^{r}\left(t\right)$ has been normalized to the interval [0,1], this paper sets $\theta_{r}$ of all resource dimensions to 0.5, which represents the middle level of the normalized resource occupation range. $\xi_{v}\left(t\right)$ is a uniformly distributed random variable satisfying $\xi_{v}\left(t\right)∼U\left(0,1\right)$, and η is the disturbance coefficient, which is set to 0.1 in this paper. The random disturbance term is used to avoid fixed selection results when multiple VMs have similar expected values, thereby enhancing the exploration capability of the search process.

After obtaining $E_{v}\left(t\right)$, the probability that VM *v* is selected by the target DC is calculated by combining the pheromone concentration between the VM and the DC:

(26) $$Prob_{v,DC}\left(t\right)=\frac{E_{v}\left(t\right)\tau_{v,DC}\left(t\right)}{\sum_{u\in \Omega_{DC}\left(t\right)}E_{u}\left(t\right)\tau_{u,DC}\left(t\right)}$$

When the denominator is zero or the pheromone intensities of all candidate VMs are unavailable, the algorithm degenerates into uniform random selection within the feasible set $\Omega_{DC}\left(t\right)$ to ensure the executability of the selection process.

The pseudo-code of the RRW VM selection mechanism is shown in Algorithm 2.
**Algorithm 2** RRW VM Selection Mechanism.**Input:** 
$\Omega_{DC}\left(t\right)$: feasible VM set for target data center DC; τ: pheromone matrix; R: set of resource types; $w_{r}$: resource weight; $\theta_{r}$: equilibrium utilization reference; η: random disturbance coefficient.**Output:** 
selectedVM: selected VM.1:  **if** $\Omega_{DC}\left(t\right)=Ø$ **then**2:       **return** null3:  **end if**4:  **for** each $v\in \Omega_{DC}\left(t\right)$ **do**5:     Generate a random disturbance variable:6:     $\xi_{v}\left(t\right)∼U\left(0,1\right)$7:     Calculate the expected value $E_{v}\left(t\right)$ using Equation (25):8:     $E_{v}\left(t\right)\leftarrow \sum_{r\in R}w_{r}\left(1-|\theta_{r}-R_{v}^{r}\left(t\right)|\right)+\eta \xi_{v}\left(t\right)$9:  **end for**10:Calculate the probability normalization term:11:$Z\leftarrow \sum_{u\in \Omega_{DC}\left(t\right)}E_{u}\left(t\right)\tau_{u,DC}\left(t\right)$12:**if** Z=0 **then**13:    Uniformly select one VM from $\Omega_{DC}\left(t\right)$ as selectedVM14:    **return** selectedVM15:**end if**16:**for** each $v\in \Omega_{DC}\left(t\right)$ **do**17:    Calculate the selection probability $Prob_{v,DC}\left(t\right)$ using Equation (26):18:    $Prob_{v,DC}\left(t\right)\leftarrow \frac{E_{v}\left(t\right)\tau_{v,DC}\left(t\right)}{Z}$19:**end for**20:$S_{prob}\leftarrow 0$21:Generate a roulette-wheel random number:22:q∼U(0,1)23:**for** each $v\in \Omega_{DC}\left(t\right)$ **do**24:    $S_{prob}\leftarrow S_{prob}+Prob_{v,DC}\left(t\right)$25:    **if** $q\leq S_{prob}$ **then**26:        **return** *v*27:    **end if**28:**end for**29:**return** the last VM in $\Omega_{DC}\left(t\right)$

#### 3.2.2. Multi-Factor Equilibrium VM Placement Process

This subsection introduces the multi-factor equilibrium ant colony optimization VM placement process based on RRW selection. In the implementation of this paper, each iteration constructs a complete VM-to-DC placement mapping that satisfies dynamic power constraints and multi-dimensional resource constraints. The algorithm first initializes the pheromone matrix τ between VMs and DCs and the pheromone evaporation rate ρ. It then traverses the candidate target DC set selectedDCs and constructs the feasible VM set $\Omega_{DC}\left(t\right)$ according to the current DC state. Within each target DC, the algorithm uses the RRW mechanism to select VMs to be placed from the feasible set. After each successful placement, it updates the power state, resource occupation state, and unassigned VM set of the target DC. After the mapping construction in each iteration is completed, the algorithm evaluates the quality of the placement scheme based on four types of normalized equilibrium factors, including load balancing, power consumption, warning-line spacing, and SLA violations, and then updates the global pheromone matrix accordingly. After multiple iterations, the algorithm retains the placement mapping with the best fitness as the final result.

The load balancing factor N(t) is calculated as:

(27) $$N\left(t\right)=\sum_{r\in R}w_{r}\left(\frac{1}{k}\sum_{j\in DClist}\left|W_{j}^{r}\left(t\right)-W_{avg}^{r}\left(t\right)\right|\right)$$

where *k* denotes the total number of DCs, $W_{j}^{r}\left(t\right)$ denotes the utilization of resource *r* in DC *j* at time *t*, $W_{avg}^{r}\left(t\right)$ denotes the average utilization of resource *r* across all DCs at time *t*, and $w_{r}$ denotes the weight assigned to resource *r*.

The power constraint utilization factor M(t) is calculated as:

(28) $$M\left(t\right)=\frac{1}{k}\sum_{j\in DClist}\frac{P_{DC_{j}}\left(t\right)}{LP_{j}\left(t\right)},$$

where $LP_{j}\left(t\right)$ denotes the dynamic hard power upper bound of DC *j* at time *t*.

The constraint boundary spacing factor A(t) is calculated as:

(29) $$A\left(t\right)=\frac{1}{k}\sum_{j\in DClist}\left[\left|\frac{P_{DC_{j}}\left(t\right)-LP_{j}\left(t\right)}{LP_{j}\left(t\right)}\right|+\sum_{r\in R}w_{r}\left|\frac{\sum_{v\in DC_{j}}V_{alloc,v}^{r}\left(t\right)-R_{j}^{r}\left(t\right)}{R_{j}^{r}\left(t\right)}\right|\right]$$

where $LP_{j}\left(t\right)$ denotes the dynamic power limit of DC *j* at time *t*, $R_{j}^{r}\left(t\right)$ denotes the physical resource capacity of DC *j* in resource dimension *r*, and $\sum_{v\in DC_{j}}V_{alloc,v}^{r}\left(t\right)$ denotes the total occupation of the VMs currently assigned to DC *j* in resource dimension *r*. The power deviation is normalized by the dynamic power limit $LP_{j}\left(t\right)$, and the resource deviation is normalized by the corresponding resource capacity $R_{j}^{r}\left(t\right)$.

The SLA violation equilibrium factor $F_{slav}\left(t\right)$ is calculated as:

(30) $$H_{slav,j}\left(t\right)=\sum_{t^{\prime }\in History}SLAv_{DC_{j}}\left(t^{\prime }\right)$$

(31) $$F_{slav}\left(t\right)=\frac{1}{k}\sum_{j\in DClist}\left|H_{slav,j}\left(t\right)-H_{avg}\left(t\right)\right|$$

where History denotes the historical scheduling time windows, $H_{slav,j}\left(t\right)$ denotes the cumulative SLA violation degree of DC *j* within the historical window, and $H_{avg}\left(t\right)$ denotes the average cumulative SLA violation degree across all DCs.

To further avoid bias in the pheromone update process caused by different numerical scales of the equilibrium factors, this paper performs candidate-scheme-level normalization on N(t), M(t), A(t), and $F_{slav}\left(t\right)$. For any equilibrium factor $Y\left(t\right)\in N\left(t\right),M\left(t\right),A\left(t\right),F_{slav}\left(t\right)$, its normalized form is:

(32) $$\hat{Y}\left(t\right)=\frac{Y(t)}{Y^{ref}\left(t\right)}$$

where $Y^{ref}\left(t\right)$ denotes the maximum reference scale of factor Y(t) in the candidate placement scheme set of the current scheduling period, defined as:

(33) $$Y^{ref}\left(t\right)=\max_{M_{m}\in M_{cand}\left(t\right)}Y_{m}\left(t\right)$$

where $M_{cand}\left(t\right)$ denotes the set of candidate placement schemes generated by MFEACO search in the current scheduling period, $M_{m}$ denotes the *m*-th candidate placement scheme, and $Y_{m}\left(t\right)$ denotes the value of N(t), M(t), A(t), or $F_{slav}\left(t\right)$ corresponding to candidate scheme $M_{m}$. After this processing, the four types of equilibrium factors are converted into comparable dimensionless quantities, thereby preventing a factor with a larger numerical range from dominating the pheromone update.

Based on the normalized equilibrium factors, the pheromone increment is defined as:

(34) $$∆\tau_{v,DC}\left(t\right)=\frac{1}{\hat{N}\left(t\right)+\hat{M}\left(t\right)+\hat{A}\left(t\right)+{\hat{F}}_{slav}\left(t\right)}$$

where $∆\tau_{v,DC}\left(t\right)$ denotes the pheromone increment corresponding to placing VM *v* into DC DC at time *t*. $\hat{N}\left(t\right)$, $\hat{M}\left(t\right)$, $\hat{A}\left(t\right)$, and ${\hat{F}}_{slav}\left(t\right)$ denote the normalized load balancing factor, power consumption factor, warning-line spacing equilibrium factor, and SLA violation equilibrium factor, respectively.

Then, the algorithm uses $∆\tau_{v,DC}\left(t\right)$ to update the global pheromone matrix $\tau_{v,DC}$. The updated global pheromone can serve as a reference for subsequent VM placement by the ant colony, while the colony continuously optimizes the placement scheme through multiple iterations until the candidate placement scheme with the best comprehensive fitness under the current iteration condition is obtained. To avoid repeated assignment of the same VM within one iteration, this paper maintains a global unassigned set during the construction of the placement mapping in each iteration, and removes a VM from this set after it is successfully placed. The pseudo-code of the Multi-Factor Equilibrium Ant Colony Optimization (MFEACO) VM placement algorithm is shown in Algorithm 3.
**Algorithm 3** MFEACO VM Placement Algorithm**Input:** 
vmsToMigrate: set of VMs to be migrated; selectedDCs: candidate target DC set; DClist: data center set; R: set of resource types; *I*: maximum number of iterations; ρ: pheromone evaporation rate; $w_{r}$: resource weight; $\theta_{r}$: equilibrium utilization reference; η: random disturbance coefficient.**Output:** 
$M_{opt}$: optimal VM placement mapping.1:  $M_{opt}\leftarrow Ø$, $Fit_{opt}\leftarrow +\infty$2:  Initialize the VM–DC pheromone matrix τ3:  Initialize the candidate solution set $M_{cand}\left(t\right)\leftarrow Ø$4:  **for**    iter=1 to *I* **do**5:     $M_{temp}\leftarrow Ø$, U←vmsToMigrate, $\tau^{\prime }\leftarrow \tau$6:     Initialize temporary DC states for the current iteration7:     **for** each DC∈selectedDCs **do**8:          **while** U≠Ø **do**9:             Construct the feasible VM set $\Omega_{DC}\left(t\right)$ from *U* using Equation (24)10:           **if** $\Omega_{DC}\left(t\right)=Ø$ **then**11:              **break**12:           **end if**13:           $selectedVM\leftarrow RRW(\Omega_{DC}\left(t\right),\tau^{\prime },DC,R,w_{r},\theta_{r},\eta )$14:           **if** selectedVM=null **then**15:              **break**16:           **end if**17:           Temporarily place selectedVM into target data center DC18:           Update the power state and resource utilization state of DC19:           $M_{temp}\leftarrow M_{temp}\cup \left\{\left(selectedVM,DC\right)\right\}$20:           U←U∖{selectedVM}21:           $\tau_{selectedVM,DC}^{\prime }\leftarrow \left(1-\rho \right)\tau_{selectedVM,DC}^{\prime }$22:        **end while**23:   **end for**24:   **if** U≠Ø **then**25:        **continue**26:   **end if**27:   $M_{cand}\left(t\right)\leftarrow M_{cand}\left(t\right)\cup \left\{M_{temp}\right\}$28:   Calculate N(t), M(t), A(t), and $F_{slav}\left(t\right)$ using Equations (27)–(31)29:   Normalize the equilibrium factors using Equations (32) and (33)30:   $Fit\left(M_{temp}\right)\leftarrow \hat{N}\left(t\right)+\hat{M}\left(t\right)+\hat{A}\left(t\right)+{\hat{F}}_{slav}\left(t\right)$31:   **for** each $\left(v,DC\right)\in M_{temp}$ **do**32:        Calculate $\ldots \tau_{v,DC}\left(t\right)$ using Equation (34)33:        $\tau_{v,DC}\leftarrow \left(1-\rho \right)\tau_{v,DC}^{\prime }+\rho \ldots \tau_{v,DC}\left(t\right)$34:   **end for**35:   **if** $Fit\left(M_{temp}\right)<Fit_{opt}$ **then**36:        $M_{opt}\leftarrow M_{temp}$37:        $Fit_{opt}\leftarrow Fit\left(M_{temp}\right)$38:   **end if**39:**end for**40:**return** $M_{opt}$

## 4. Experimental Evaluation and Performance Analysis

To verify the scheduling performance of the proposed two-stage VM migration framework under dynamic power constraints, this paper conducts experimental evaluation based on an improved CloudSim simulation platform [28] and two real-world cluster trace datasets from Google [29] and Alibaba [30]. The experiments mainly focus on the following questions. First, whether the proposed method can keep DC energy consumption within the constraint boundary during the variation of dynamic power limits. Second, whether the MFB VM selection mechanism and the MFEACO VM placement mechanism can improve energy consumption, SLA violations, and the number of migrations under different workload datasets. Third, under a fixed VM selection strategy, whether MFEACO achieves more stable multi-objective trade-off performance than traditional heuristic methods and metaheuristic methods. Fourth, whether the proposed algorithms can satisfy the real-time or near-real-time scheduling requirements under dynamic power constraints in terms of asymptotic complexity and actual execution time.

### 4.1. Experimental Setup

#### 4.1.1. Simulation Platform and Test System Configuration

This paper extends the CloudSim simulation platform to support VM migration, dynamic power constraints, and multi-metric scheduling evaluation in multi-DC scenarios. The experimental system contains 30 geographically distributed DCs, and time-varying power upper bounds are assigned to some of them to simulate constrained power supply scenarios caused by the combined effects of grid supply capability variations, renewable energy output fluctuations, and demand response mechanisms.

For host-side configuration, this paper adopts a DC-level equivalent resource pool modeling approach. The available host-side resources are aggregated and represented as four types of equivalent resource capacities at the DC level, namely CPU, memory, disk I/O, and network I/O. During scheduling, migrated VMs must simultaneously satisfy the resource capacity constraints and the dynamic power upper-bound constraints of the target DC. For VM configuration, the number of VMs and their resource demands are mapped from task-level trace data in the Google Cluster Trace and Alibaba Cluster Trace. The original task records are divided into time windows with a 5 min scheduling interval, and active tasks within each scheduling window are mapped into VM load units. Each VM has four types of resource demands in the corresponding scheduling period, including CPU, memory, disk I/O, and network I/O. The CPU and memory demands are obtained by normalizing the resource usage records in the traces. For disk I/O and network I/O, which are not completely provided or have inconsistent sampling granularity in the original traces, proxy load sequences are constructed according to the task active state and normalized computing load intensity to represent the relative pressure on disk and network resources.

In the initialization stage, all VMs are assigned to the 30 DCs according to a unified rule. A round-robin strategy is adopted to generate the initial VM-to-DC mapping, thereby avoiding artificial bias toward any specific DC in the initial distribution. For the same dataset, all comparison algorithms use the same initial VM distribution, the same task arrival sequence, the same VM resource demand sequence, and the same dynamic power constraint curves.

The simulation period is set to 24 h, and the scheduling interval is 5 min. Therefore, each group of experiments contains 288 scheduling periods. In each scheduling period, the system determines whether migration should be triggered according to the current VM load state, DC resource utilization state, and dynamic power constraints, and then performs migration candidate VM selection and target DC placement decisions.

All experiments are conducted on a workstation equipped with an Intel Core i7-12700 processor, 32 GB memory, and the Windows 11 operating system. The experimental platform is based on Java 1.8 and the improved CloudSim simulation platform. The algorithm execution time is recorded using System.nanoTime(), and the statistical scope only includes the scheduling decision process itself, excluding additional overheads such as data reading, result saving, file I/O, and figure drawing.

#### 4.1.2. Datasets and Workload Construction

This paper uses two public real-world cluster trace datasets, namely Google Cluster Trace and Alibaba Cluster Trace, to generate VM workloads. Both datasets record information such as task submission, running states, and resource usage variations in large-scale cluster environments, and can reflect the time-varying characteristics of task arrivals and resource demands in real cloud DCs. Google Cluster Trace is mainly used to construct a relatively stable large-scale cloud DC workload scenario, while Alibaba Cluster Trace contains more concentrated task submissions and resource demand variations, and is therefore used to construct a highly dynamic test scenario with more obvious workload fluctuations. By using both trace datasets, this paper evaluates the adaptability of the proposed scheduling framework under different workload fluctuation conditions.

For workload construction, this paper first extracts fields such as task arrival time, running duration, CPU usage, and memory usage from the original traces, and aggregates the active tasks within time windows according to the 5 min scheduling interval. If a task is running in scheduling window *t*, its resource usage is counted into the VM load corresponding to that window. If multiple tasks are mapped to the same VM load unit, their resource demands are accumulated. Through this process, the original task traces are transformed into VM resource demand sequences over discrete scheduling periods.

For data preprocessing, records with missing key fields, non-positive running durations, or out-of-range resource usage values are removed. For extremely abnormal resource usage values, boundary clipping is adopted to limit them within a reasonable range, thereby reducing the impact of abnormal samples on the simulation results. Then, the resource demands are normalized according to the DC resource capacities, so that the workloads under different datasets and different resource dimensions are uniformly mapped to the interval [0,1]. The normalized CPU and memory demands are directly obtained from the resource usage records in the traces.

For disk I/O and network I/O resources, since the corresponding fields are not completely provided in the original trace data, this paper constructs proxy load sequences according to the task active state and normalized computing load intensity. In this way, CPU, memory, disk I/O, and network I/O can jointly participate in resource capacity constraint judgment, SLA violation calculation, and VM placement feasibility judgment. In addition, the proxy loads of disk I/O and network I/O are only used to represent relative resource pressure, and are not intended to claim that complete disk or network measurement data exist in the original traces.

Finally, to ensure fair comparison among different algorithms, all comparison algorithms under the same dataset use the same task arrival sequence, VM resource demand sequence, initial VM distribution, and dynamic power constraint curves. In other words, the performance differences in the experimental results mainly come from the VM selection strategy and VM placement strategy themselves, rather than from differences in workload input, initialization state, or power constraint settings.

#### 4.1.3. Experimental Parameter Settings

To evaluate the roles of the VM selection stage and the VM placement stage in the proposed framework, multiple algorithm combinations are selected as comparison methods. The VM selection methods include the Maximum Correlation (MC) algorithm, the Minimum Utilization (MU) algorithm, and the proposed Multi-Factor Balanced (MFB) selection algorithm. The VM placement methods include the Minimum Post-Migration Energy Increment (MPEI) algorithm, the Minimum Power Consumption Host Post-Migration (MPCHP) algorithm, the MinMax Host Utilization (MHU) algorithm, the Power-Constrained Genetic Algorithm (PC-GA), and the proposed Multi-Factor Equilibrium Ant Colony Optimization (MFEACO) algorithm.

Among them, MPEI, MPCHP, and MHU are used to represent typical heuristic VM placement strategies, while PC-GA is used to represent a typical metaheuristic search strategy. PC-GA encodes the mapping relationship between VMs and target DCs as chromosomes, and its fitness function jointly considers energy consumption, SLA violations, the number of migrations, and penalty terms for power and resource constraints. By comparing heuristic placement strategies, genetic search strategies, and the proposed MFEACO strategy, the scheduling performance differences among different placement mechanisms under dynamic power constraints can be evaluated more comprehensively.

Table 1 presents the main algorithm parameters and random experiment settings. To reduce the influence of random initialization on the experimental results, all algorithms involving random selection use the same random seeds and are executed under the same initial VM distribution, the same dynamic power constraints, and the same workload input. Each group of experiments is repeated 10 times, and the average results are reported.

To further evaluate the computational feasibility of the proposed method, this paper records the actual execution time of the MFEACO placement process in each scheduling interval over the 24 h simulation period. The runtime statistics only include the algorithm scheduling decision process itself, excluding additional overheads such as data reading, result saving, file I/O, and figure drawing.

### 4.2. Results and Discussion

Based on the above experimental settings, this paper evaluates the proposed MFB–MFEACO framework from five aspects. First, it analyzes whether typical DCs can continuously satisfy dynamic power constraints over the 24 h scheduling period, so as to verify the scheduling feasibility of the proposed method under constrained power scenarios. Second, it compares the comprehensive performance of different VM selection and placement strategy combinations on the Google and Alibaba real-world cluster datasets, so as to evaluate the adaptability of the proposed two-stage framework under different workload characteristics. Third, under the fixed MFB selection strategy, it further compares the scheduling results of different placement algorithms, highlighting the performance differences between the MFEACO placement mechanism and traditional heuristic methods as well as the PC-GA metaheuristic method. Fourth, it analyzes the comprehensive trade-off among energy consumption, SLA violations, and the number of migrations through a normalized multi-objective heatmap. Finally, it analyzes the computational efficiency of the proposed algorithms from three aspects, including asymptotic complexity, actual execution time, and convergence behavior, to verify their real-time feasibility within the scheduling period under dynamic power constraints.

#### 4.2.1. Feasibility Analysis Under Dynamic Power Constraints

**Figure 2:**
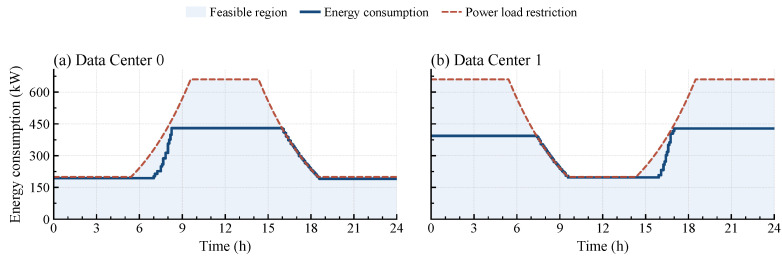
Operating power variation and feasible constraint range of typical DCs under dynamic power limits.

**Figure 3:**
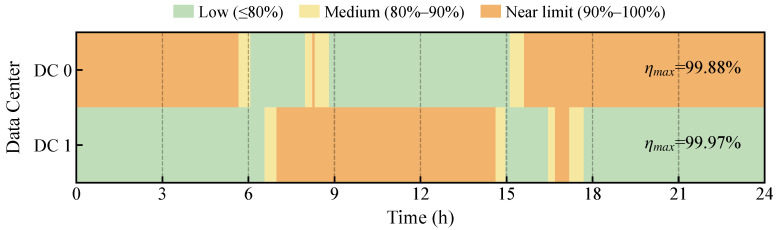
Distribution of dynamic power constraint utilization states in typical DCs.

In constrained power load scenarios, the scheduling results must first satisfy the dynamic power upper-bound constraints of DCs. If the operating power of a DC after scheduling exceeds the power upper bound, the scheduling scheme is not practically feasible even if it performs well in terms of energy consumption or SLA metrics. Therefore, this subsection first selects two typical constrained DCs and analyzes the compliance of the proposed MFB–MFEACO framework with dynamic power constraints over the 24 h scheduling period. The experimental sampling interval is 5 min, including 288 scheduling periods in total. Figure 2 shows the operating power variation and feasible constraint range of two typical DCs under dynamic power limits, and Figure 3 shows the corresponding distribution of power constraint utilization states.

As shown in Figure 2, the dashed lines represent the dynamic power upper bounds of the DCs at different time points, the solid lines represent the actual operating power after scheduling, and the filled regions represent the feasible operating intervals determined by the power constraints. It can be observed that DC 0 and DC 1 do not exceed their dynamic power upper bounds throughout the 24 h scheduling period, indicating that the proposed framework can maintain constraint feasibility under different power limit variation conditions.

Further observation of the constraint variation processes of the two DCs shows that DC 0 and DC 1 face different temporal characteristics of power limits. The available power capacity of DC 0 first becomes tight, then relaxes, and finally tightens again, while the available power capacity of DC 1 first remains relatively loose, then tightens, and finally recovers. During the constraint tightening stage, the operating power after scheduling decreases with the falling power boundary. During the constraint relaxation stage, the operating power can moderately increase within the feasible range. This indicates that VM migration scheduling is not merely a static adjustment under a fixed power threshold, but can update the load distribution according to dynamic power boundary variations.

Figure 3 further shows the operating states of the two typical DCs from the perspective of constraint utilization. In this paper, power constraint utilization is divided into four categories: relaxed state (≤80%), moderate state (80–90%), near-constraint state (90–100%), and violation state (>100%). Figure 3 displays only the first three non-violation states because no violation state occurs during the scheduling period. Specifically, the maximum constraint utilization of DC 0 is 99.88%, and that of DC 1 is 99.97%; both values are close to but do not exceed the dynamic power upper bounds.

The above results demonstrate that the proposed MFB–MFEACO framework can maintain power constraint feasibility in typical constrained DCs, and can keep power utilization close to the constraint boundary in some periods. This provides the premise for the subsequent comparative analysis of relative energy increase, SLA violations, and the number of migrations: all subsequent performance comparisons are conducted under the condition that dynamic power constraints are satisfied, rather than by obtaining lower energy consumption or fewer migrations at the cost of violating power upper bounds.

#### 4.2.2. Performance Comparison of Different VM Selection and Placement Strategy Combinations

**Figure 4:**
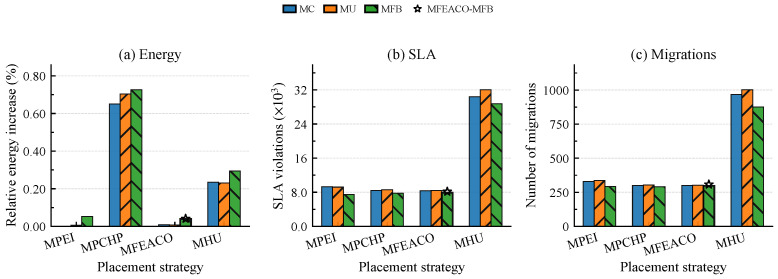
Comprehensive performance comparison of different scheduling strategies on the Google dataset.

**Figure 5:**
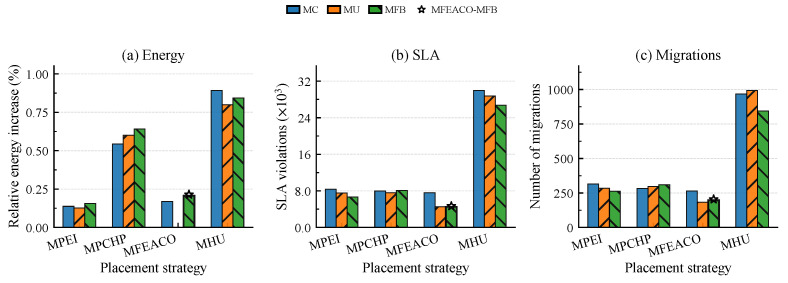
Comprehensive performance comparison of different scheduling strategies on the Alibaba dataset.

On the basis of verifying the feasibility under dynamic power constraints, this paper further compares the comprehensive scheduling performance of different VM selection and placement strategy combinations. This group of experiments changes both the VM selection algorithm and the VM placement algorithm, and is used to evaluate the impact of different two-stage scheduling combinations on relative energy increase, the number of SLA violations, and the number of VM migrations. Figure 4 and Figure 5 present the comparison results on the Google dataset and the Alibaba dataset, respectively. The relative energy increase is normalized with respect to the lowest energy consumption result within the corresponding dataset, so that the relative differences among different algorithms can be observed more clearly.

As shown in Figure 4, under the relatively stable workload scenario corresponding to the Google dataset, different selection–placement combinations show obvious differences in the number of SLA violations and the number of VM migrations. Host-utilization-driven placement strategies represented by MHU produce relatively high numbers of SLA violations and migrations in multiple combinations, indicating that placement based solely on host utilization is difficult to fully adapt to the multi-objective scheduling requirements under dynamic power constraints. By contrast, MFEACO-related combinations generally maintain lower SLA violations and fewer migrations, showing that jointly considering energy consumption, load balancing, constraint boundaries, and historical SLA states in the placement stage helps improve the stability of scheduling results.

Further observation of the VM selection strategies shows that, compared with MC and MU, MFB-related combinations are more stable in most cases in terms of SLA violations and VM migrations. This indicates that MFB does not simply select VMs with the highest or lowest resource occupation. Instead, it jointly screens migration objects according to the power warning line trend, resource warning line trend, and SLA violation degree, thereby improving the quality of the candidate set for the subsequent placement stage. It should be noted that different selection strategies still show certain fluctuations in the single metric of relative energy increase. Therefore, the advantage of MFB is mainly reflected in suppressing service quality loss and controlling migration overhead. On the Google dataset, MFB–MFEACO maintains relatively low levels of both SLA violations and migrations, indicating good synergy between the trend-aware selection mechanism and the multi-factor equilibrium placement mechanism.

As shown in Figure 5, under the highly fluctuating workload scenario corresponding to the Alibaba dataset, the performance differences among different algorithm combinations become more obvious. Since the Alibaba workload has stronger time-varying and bursty characteristics, the scheduling algorithms need to make trade-offs between more frequent workload changes and tighter power constraints. The experimental results show that MHU-related combinations still tend to produce relatively high numbers of SLA violations and migrations, indicating that placement based only on host utilization lacks robustness under complex workload fluctuations. MPEI- and MPCHP-related combinations can control the relative energy increase to some extent, but still show insufficient trade-off performance in terms of SLA violations or the number of migrations.

By contrast, MFB–MFEACO still maintains relatively low relative energy increase, SLA violations, and VM migrations on the Alibaba dataset. This result indicates that the MFB stage can screen more reasonable migration objects according to dynamic constraint variations, avoiding blind migration or delayed migration. Meanwhile, the MFEACO stage can perform multi-factor equilibrium search among candidate DCs, avoiding placement decisions that are overly biased toward a single energy consumption objective or a single resource utilization objective. Therefore, the proposed two-stage framework still shows good adaptability in scenarios with stronger workload fluctuations.

The experimental results on both Google and Alibaba datasets show that the advantage of the proposed MFB–MFEACO framework does not come from a single stage alone, but from the synergy between VM selection and VM placement. MFB improves the effectiveness of the migration candidate set through the trend-aware mechanism, while MFEACO improves the quality of target DC selection through multi-factor equilibrium search. Compared with traditional heuristic combinations, the proposed two-stage combination achieves more stable comprehensive performance in terms of relative energy increase, SLA violation suppression, and migration overhead control while satisfying dynamic power constraints.

#### 4.2.3. Placement Strategy Comparison Under Fixed MFB

**Figure 6:**
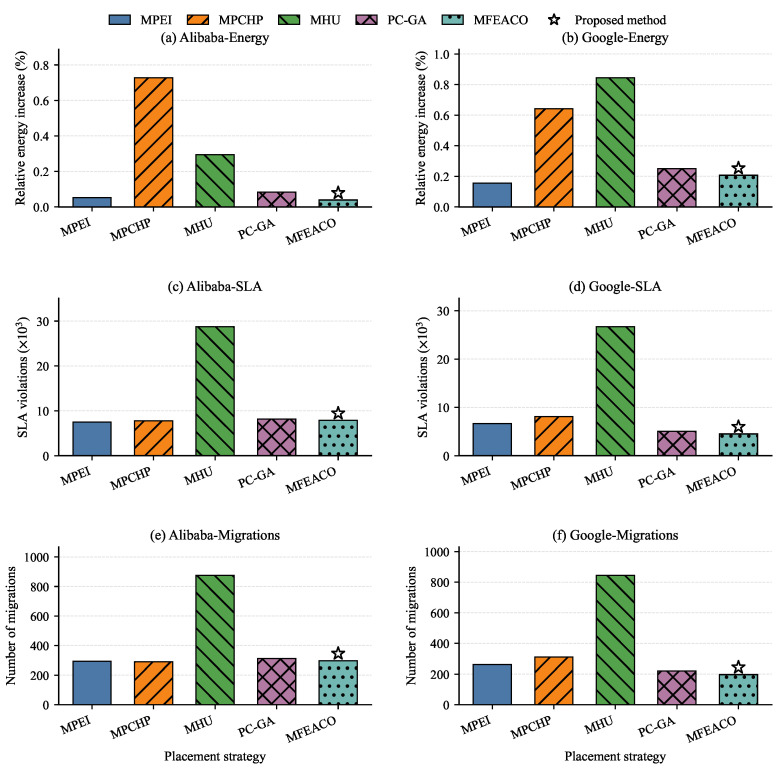
Performance analysis of different placement algorithms under the fixed MFB selection strategy.

The previous subsection verifies the overall effectiveness of the MFB–MFEACO framework from the perspective of algorithm combinations. To further distinguish the influence of the VM selection stage and the VM placement stage on the experimental results, this subsection fixes MFB as the VM selection strategy, changes only the VM placement algorithm, and compares the scheduling results of five placement strategies, namely MPEI, MPCHP, MHU, PC-GA, and MFEACO. Through this controlled-variable setting, the role of the MFEACO placement mechanism under dynamic power constraints can be analyzed more directly. Figure 6 shows the relative energy increase, the number of SLA violations, and the number of VM migrations of different placement algorithms on the Google and Alibaba datasets under the fixed MFB selection strategy.

On the Google dataset, the relative energy increase, number of SLA violations, and number of migrations of MFB–MFEACO are 0.207%, $4.51\times 10^{3}$, and 197, respectively. Compared with MFB–PC-GA, MFB–MFEACO reduces the relative energy increase, the number of SLA violations, and the number of migrations by approximately 17.2%, 10.7%, and 10.5%, respectively. This indicates that, under a relatively stable workload scenario, MFEACO does not reduce energy consumption simply by increasing the number of migrations. Instead, it controls service quality loss and migration overhead while reducing the energy increase.

Compared with traditional heuristic placement algorithms, MFB–MFEACO also shows better comprehensive performance on the Google dataset. MPEI pays more attention to the energy increment after migration, so its energy metric is relatively low, but it is inferior to MFEACO in terms of SLA violations and the number of migrations. MPCHP and MHU are biased toward the minimum-power host and host utilization balancing, respectively. However, such local criteria are difficult to simultaneously characterize the relationships among power constraints, load balancing, and historical SLA states. By contrast, MFEACO comprehensively considers load balancing, power constraint margin, warning-line distance, and SLA violation differences through the multi-factor pheromone update mechanism, thereby forming a more stable trade-off among multiple metrics.

On the Alibaba dataset, the relative energy increase, number of SLA violations, and number of migrations of MFB–MFEACO are 0.039%, $7.87\times 10^{3}$, and 297, respectively. Compared with MFB–PC-GA, MFB–MFEACO reduces the relative energy increase from 0.083% to 0.039%, the number of SLA violations from $8.15\times 10^{3}$ to $7.87\times 10^{3}$, and the number of migrations from 312 to 297. This result shows that, in a scenario with stronger workload fluctuations, MFEACO can still maintain a lower energy increase than PC-GA, while achieving certain improvements in SLA violations and the number of migrations.

It should be noted that, on the Alibaba dataset, MFB–MFEACO is not absolutely optimal in every single metric. For example, MFB–MPEI achieves a slightly lower number of SLA violations, and MFB–MPCHP has a slightly smaller number of migrations. However, MFB–MFEACO obtains the lowest relative energy increase, while keeping the numbers of SLA violations and migrations at relatively low levels. Compared with MFB–MHU, MFB–MFEACO reduces the number of SLA violations and the number of migrations by approximately 72.6% and 66.1%, respectively. This indicates that, under highly fluctuating workloads and stronger dynamic power constraints, the main advantage of MFEACO lies in its comprehensive multi-objective trade-off capability rather than extreme optimization of a single metric.

In summary, under the fixed MFB selection strategy, MFEACO exhibits more stable comprehensive scheduling performance than traditional heuristic placement strategies and the PC-GA metaheuristic placement strategy. This result further demonstrates that the multi-factor equilibrium pheromone update mechanism in MFEACO can improve the quality of VM placement decisions, enabling the scheduling results to maintain a good balance among energy consumption control, SLA violation suppression, and migration overhead control.

#### 4.2.4. Multi-Objective Scheduling Trade-Off Analysis

The preceding experiments compare different placement algorithms in terms of three metrics: relative energy increase, the number of SLA violations, and the number of VM migrations. However, these three metrics have different dimensions and numerical ranges, making it difficult to directly judge the comprehensive performance of algorithms in multi-objective scenarios according to raw values. To further analyze the trade-off relationships among multiple objectives for different placement algorithms, this paper normalizes each performance metric under the fixed MFB selection strategy and draws multi-objective performance heatmaps.

**Figure 7:**
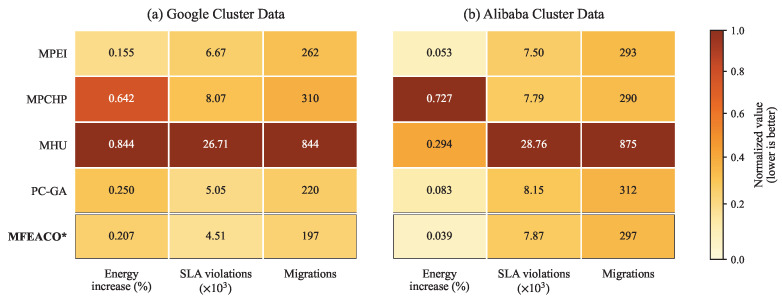
Multi-objective performance heatmaps of different placement algorithms under the fixed MFB selection strategy. MFEACO* denotes the proposed MFB–MFEACO combination.

For each dataset and each evaluation metric, this paper adopts the following normalization method:

(35) $${\hat{x}}_{a,m}=\frac{x_{a,m}}{\max_{a\in A}x_{a,m}}$$

where $x_{a,m}$ denotes the original result of algorithm *a* on metric *m*, A denotes the set of placement algorithms involved in the comparison, and ${\hat{x}}_{a,m}$ denotes the normalized metric value. Since the relative energy increase, the number of SLA violations, and the number of VM migrations considered in this paper are all cost-type metrics, a smaller normalized value indicates better performance on the corresponding metric. Figure 7 shows the multi-objective performance heatmaps of different placement algorithms on the Google and Alibaba datasets under the fixed MFB selection strategy. Figure 7a corresponds to the Google dataset, and Figure 7b corresponds to the Alibaba dataset. The colors represent the normalized cost metrics, where lighter colors indicate better performance, and the values in the cells are the original results of the corresponding metrics.

As shown in Figure 7, MFEACO does not show obvious deterioration in any metric on either dataset, and can generally maintain a good trade-off among relative energy increase, the number of SLA violations, and the number of migrations. By contrast, heuristic methods such as MPEI, MPCHP, and MHU usually show advantages only in some individual metrics. Although PC-GA performs relatively evenly, its comprehensive trade-off effect is still weaker than that of MFEACO. This result indicates that the proposed MFEACO placement strategy can improve placement stability under dynamic power constraints through multi-factor equilibrium search.

#### 4.2.5. Computational Efficiency and Real-Time Feasibility Analysis

In addition to performance metrics such as energy consumption, the number of SLA violations, and the number of migrations, DC load scheduling under dynamic power constraints also needs to consider the computational efficiency of the algorithm, since the scheduling algorithm must complete migration decisions within a limited control period. Therefore, this subsection first analyzes the time and space complexity of the three core algorithms proposed in this paper, and then further records the actual execution time and convergence behavior of the MFEACO algorithm over the 24 h simulation period to verify its feasibility for real-time or near-real-time scheduling.

Let *N* denote the total number of VMs in the system, *K* denote the number of migration candidate VMs after MFB screening, *C* denote the number of candidate target DCs, *R* denote the number of resource types, and *I* denote the maximum number of MFEACO iterations. This paper considers four types of resources, namely CPU, memory, disk I/O, and network I/O. Therefore, R=4 and can be regarded as a constant. In general, only some VMs in power-constrained or resource-stressed DCs enter the migration candidate set. Therefore, *K* is usually much smaller than the total number of VMs *N*.

For Algorithm 1, the MFB candidate VM screening algorithm mainly scores candidate VMs in power-constrained or resource-stressed DCs. For each candidate VM, the algorithm needs to calculate the SLA violation degree, power warning line trend, resource warning line trend, VM power consumption contribution, and comprehensive migration priority score. The resource-related terms need to traverse *R* resource dimensions. Therefore, the time complexity of Algorithm 1 is O(KR). Since R=4 in this paper, the complexity can be simplified to O(K). Its space complexity mainly comes from storing the candidate VM set and scoring results, and is therefore O(K).

For Algorithm 2, the RRW VM selection mechanism first calculates the expectation value of each candidate VM in the feasible VM set, then calculates the normalized selection probability by combining the pheromone concentration, and finally completes roulette-wheel selection through cumulative probability. Since the denominator of probability normalization only needs to be calculated once in advance and can be reused in subsequent probability calculations, the time complexity of Algorithm 2 is O(KR+K), which can be simplified to O(K). If the expectation value and selection probability of each VM are explicitly stored, the space complexity is O(K).

For Algorithm 3, the MFEACO VM placement algorithm needs to perform iterative ant colony search among multiple candidate target DCs. In each iteration, the algorithm traverses the candidate target DCs and selects feasible VMs from the migration candidate set for placement based on the RRW mechanism. As the placement process proceeds, the number of unassigned VMs gradually decreases. In the worst case, each candidate DC may scan and check the feasibility of the migration candidate set. Therefore, the time complexity of a single iteration can be expressed as $O(CK^{2}R)$. Considering the maximum number of iterations *I*, the overall time complexity of Algorithm 3 is $O(ICK^{2}R)$. Since *R* is a fixed constant, this complexity can be simplified to $O(ICK^{2})$. The space complexity of Algorithm 3 is mainly determined by the DC pheromone matrix and the temporary placement mapping, and is therefore O(KC).

Table 2 summarizes the asymptotic time complexity of the three algorithms. It can be seen that Algorithms 1 and 2 both have linear complexity, and their main computational overhead increases with the number of migration candidate VMs. Algorithm 3 has relatively higher complexity because this stage requires iterative combinatorial search between candidate VMs and candidate DCs. However, unlike global re-placement of all *N* VMs, the proposed two-stage framework restricts the search objects to the *K* migration candidate VMs screened by MFB, thereby avoiding exponential combinatorial search in the full-scale placement problem. Therefore, the computational advantage of the proposed framework does not lie in the lowest complexity of a single MFEACO search step, but in transforming the original large-scale scheduling problem into a smaller candidate-set optimization problem through the candidate screening mechanism.

On the basis of the theoretical complexity analysis, this paper further records the actual execution time and convergence behavior of the MFEACO algorithm over the 24 h simulation period. Since the experiment adopts 5 min as one scheduling interval, each dataset contains 288 scheduling decision moments. The runtime statistics only include the algorithm scheduling decision process itself, excluding additional overheads such as data reading, result saving, file I/O, and figure drawing. In this paper, the number of convergence iterations is defined as the first iteration number at which the current best placement objective value shows no improvement for five consecutive iterations. If this condition is not satisfied within the maximum number of iterations *I*, then *I* is taken as the number of convergence iterations for that scheduling interval.

As shown in Table 3, on the Google Cluster dataset, the average execution time of the MFEACO algorithm is 1.26 s, and the maximum execution time is 2.91 s. On the Alibaba Cluster dataset, the average execution time is 1.38 s, and the maximum execution time is 3.16 s. These execution times are significantly lower than the 300 s scheduling interval adopted in this simulation, indicating that the proposed MFEACO placement strategy can complete the scheduling decision before the next power constraint variation moment arrives.

Further observation of the convergence iterations shows that MFEACO can reach a stable solution within a relatively small number of iterations on both datasets. Specifically, the average and maximum numbers of convergence iterations on the Google Cluster dataset are 23.4 and 41, respectively, while those on the Alibaba Cluster dataset are 25.1 and 44, respectively. The convergence iterations on the Alibaba dataset are slightly higher than those on the Google dataset, mainly because the Alibaba workload fluctuates more strongly and the multi-objective relationships vary more significantly. Nevertheless, the maximum execution time on both datasets remains within the second-level range, which is far below the 5 min scheduling period.

In summary, the proposed two-stage framework first compresses the full-scale VM scheduling problem into a placement optimization problem over the candidate migration VM set through the MFB screening mechanism, and then completes multi-factor equilibrium search within a smaller search space through MFEACO. Both the theoretical complexity analysis and the actual runtime results demonstrate that the proposed method can satisfy the real-time scheduling requirements under dynamic power constraints at the current experimental scale and scheduling period.

## 5. Conclusions

To address the difficulty of collaboratively optimizing energy consumption control, service quality assurance, and migration overhead in DC information load scheduling under dynamic power constraints, this paper proposes a two-stage VM migration optimization framework. The proposed framework incorporates grid-side time-varying power upper bounds, DC operational states, and virtualization-layer workload information into a unified scheduling process, and realizes the dynamic matching between information loads and available power capacity through cross-DC VM migration. In the VM selection stage, the proposed MFB algorithm uses an arctangent-function-based warning line trend model to characterize the variation directions of power loads and resource constraints, and selects migration candidate VMs by jointly considering SLA violation degree, power consumption contribution, and resource occupation. In the VM placement stage, the proposed MFEACO algorithm employs the RRW selection mechanism and a multi-factor equilibrium pheromone update strategy to comprehensively balance power constraint utilization, load balancing, warning-line spacing, and historical SLA states, thereby improving the quality of VM placement under dynamic power constraints.

Simulation experiments based on the improved CloudSim platform and the Google Cluster Trace and Alibaba Cluster Trace show that the proposed MFB–MFEACO framework can keep the operating power of constrained DCs below the dynamic power upper bounds throughout the 24 h scheduling period. In the comparison of different VM selection and placement strategy combinations, MFB–MFEACO shows good comprehensive scheduling performance on both real-world workload datasets. Furthermore, under the fixed MFB selection strategy, the comparison among different placement algorithms shows that MFEACO achieves a more stable trade-off among relative energy increase, the number of SLA violations, and the number of migrations than traditional heuristic placement strategies and the PC-GA metaheuristic baseline. The normalized multi-objective heatmap results further indicate that the advantage of MFEACO does not rely on the extreme optimization of a single metric, but mainly lies in its comprehensive balance of multi-objective scheduling performance under dynamic power constraints.

Nevertheless, this paper still has room for further extension. The current model mainly focuses on steady-state operating power optimization and cross-DC VM placement decisions under dynamic power constraints, while the transient energy consumption, migration delay, link transmission overhead, and network congestion impact during each online migration process have not yet been finely modeled. In addition, the weight parameters, pheromone evaporation rate, and random disturbance coefficient in the proposed algorithms are still set as fixed values, and their parameter adaptivity in heterogeneous DC scenarios needs to be further improved. Future work will incorporate more fine-grained migration process models, dynamic network state awareness mechanisms, and real testbed validation to further improve the engineering applicability and robustness of the proposed framework in complex DC environments.

## Figures and Tables

**Table 1:** Algorithm parameter settings.

Category	Parameter	Value
MFEACO	Maximum number of iterations *I*	50
MFEACO	Pheromone evaporation rate ρ	0.5
RRW	Random disturbance coefficient η	0.1
RRW	Resource equilibrium reference value $\theta_{r}$	0.5
PC-GA	Population size	50
PC-GA	Maximum number of iterations	50
PC-GA	Crossover probability	0.8
PC-GA	Mutation probability	0.1
Experimental setting	Random seed set	2026–2035
Experimental setting	Number of repeated experiments	10

**Table 2:** Asymptotic complexity analysis of the proposed algorithms.

Algorithm	Time Complexity	Space Complexity
Algorithm 1: MFB screening algorithm	O(KR), simplified as O(K)	O(K)
Algorithm 2: RRW selection mechanism	O(KR+K), simplified as O(K)	O(K)
Algorithm 3: MFEACO placement algorithm	$O(ICK^{2}R)$, simplified as $O(ICK^{2})$	O(KC)

**Table 3:** Execution time and convergence behavior of MFEACO over the 24 h simulation period.

Dataset	Average Time/s	Maximum Time/s	Average Convergence Iterations	Maximum Convergence Iterations
Google Cluster	1.26	2.91	23.4	41
Alibaba Cluster	1.38	3.16	25.1	44

## Data Availability

Publicly available datasets were analyzed in this study. These data can be found here: Google Cluster Data [https://github.com/google/cluster-data (accessed on 10 June 2026)] and Alibaba Cluster Data [https://github.com/alibaba/clusterdata (accessed on 10 June 2026)].
